# Effects of nebulized ketamine on allergen-induced airway hyperresponsiveness and inflammation in actively sensitized Brown-Norway rats

**DOI:** 10.1186/1476-9255-4-10

**Published:** 2007-05-04

**Authors:** Min Min Zhu, Qin Hai Zhou, Mei Hua Zhu, Hai Bo Rong, Yu Ming Xu, Yan Ning Qian, Cheng Zhang Fu

**Affiliations:** 1 Nanjing Medical University, 140 Hanzhong Road, Nanjing, 210029, PR China; 2 Department of Anesthesiology, the First Affiliated Hospital of Nanjing Medical University, 300 Guangzhou Road, Nanjing, 210029, PR China; 3 Department of Anesthesiology, Affiliated Brain Hospital of Nanjing Medical University, 264 Guangzhou Road, Nanjing, 210029, PR China; 4 Jiangsu Institute of Anesthesiology, 99 Huaihai Road, Xuzhou, 221002, PR China

## Abstract

Since airway hyperresponsiveness (AHR) and allergic inflammatory changes are regarded as the primary manifestations of asthma, the main goals of asthma treatment are to decrease inflammation and maximize bronchodilation. These goals can be achieved with aerosol therapy. Intravenous administration of the anesthetic, ketamine, has been shown to trigger bronchial smooth muscle relaxation. Furthermore, increasing evidence suggests that the anti-inflammatory properties of ketamine may protect against lung injury. However, ketamine inhalation might yield the same or better results at higher airway and lower ketamine plasma concentrations for the treatment of asthma. Here, we studied the effect of ketamine inhalation on bronchial hyperresponsiveness and airway inflammation in a Brown-Norway rat model of ovalbumin(OVA)-induced allergic asthma. Animals were actively sensitized by subcutaneous injection of OVA and challenged by repeated intermittent (thrice weekly) exposure to aerosolized OVA for two weeks. Before challenge, the sensitizened rats received inhalation of aerosol of phosphate-buffered saline (PBS) or aerosol of ketamine or injection of ketamine respectivity. Airway reactivity to acetylcholine (Ach) was measured in vivo, and various inflammatory markers, including Th2 cytokines in bronchoalveolar lavage fluid (BALF), as well as induciable nitric oxide synthase (iNOS) and nitric oxide (NO) in lungs were examined. Our results revealed that delivery of aerosolized ketamine using an ultrasonic nebulizer markedly suppressed allergen-mediated airway hyperreactivity, airway inflammation and airway inflammatory cell infiltration into the BALF, and significantly decreased the levels of interleukin-4 (IL-4) in the BALF and expression of iNOS and the concentration of NO in the inflamed airways from OVA-treated rats. These findings collectively indicate that nebulized ketamine attenuated many of the central components of inflammatory changes and AHR in OVA-provoked experimental asthma, potentially providing a new therapeutic approach against asthma.

## Background

Asthma is characterized by acute and chronic airway inflammation, in which the severity of airway hyperreactivity (AHR) is correlated with the degree of inflammation [1]. Based on our growing understanding of asthma pathogenesis, the various treatment strategies are primarily focused on decreasing inflammation and maximizing bronchodilation. Inhalotherapy represents the main method currently used for treating respiratory disorders, because it has the benefit of allowing drugs to be delivered directly to the large surfaces of the tracheobronchial tree and alveoli [2]. Inhaled drugs play an important role in asthma management, as many of the beta-adrenergic and anti-cholinergic bronchodilators, corticosteroids, and nonsteroidal anti-inflammatory agents currently used in the treatment of acute asthma are administered as inhaled gases or aerosols [3]. However, although steroids and beta-agonists form the mainstay of asthma therapy, the symptoms in some asthmatics are poorly controlled with these drugs, and their therapeutic benefits may be outweighed to some degree by their undesirable side effects [4]. Thus, researchers continue to seek and evaluate additional drugs capable of modulating inflammatory responses and AHR in the treatment of asthma.

The intravenous anesthetic, ketamine, was first used in humans in 1965 and is still applied in a variety of clinical settings today. Ketamine has many pharmacological properties, including analgesic, anesthetic and sympathomimetic effects [5]. Owing to its ability to induce relaxation of bronchial smooth muscle, ketamine is recommended as an optimizing anesthetic for asthmatic patients, and has been clinically used to treat bronchospasms, asthma exacerbation and status asthmaticus [6-8]. In recent years, studies have shown that ketamine plays a protective role against lung injury, via its anti-inflammatory properties. For example, under ketamine anesthesia, neurogenic pulmonary edema is less pronounced in a rat model of spinal cord injury [9], and the mortality of severely burnt rats is reduced [10]. In addition, ketamine has been shown to attenuate symptoms of endotoxemia in a lipopolysaccharide (LPS)-induced rat model of of sepsis, by reducing NF-kappa B activity and TNF-alpha production [11], and decreasing the expression of inducible nitric oxide synthase (iNOS) [12], which has been implicated in endotoxin-induced tissue injury. Taken together, these results suggest that ketamine has anti-inflammatory and anti-hyperresponsiveness effects, and could prove useful for the treatment of asthma. However, the use of ketamine in asthma patients has been limited by the risk of adverse systemic effects following administration by the conventional route of intravenous injection [13]. Studies into the nasal, oral, and rectal administration of ketamine have suggest that local use of this drug is both effective and plausible [14-16]. However, no previous work has investigated the possible use of ketamine inhalation for treatment in asthma.

The aim of this investigation was to evaluate whether ketamine treatment by the inhaled route could prove efficient and safe for the treatment of asthma. We examined the effect of nebulized ketamine inhalation on allergen-induced AHR and inflammatory changes in OVA-sensitized Brown-Norway rats, a model in which chronic OVA challenge is used to stimulate airway inflammation and AHR [17]. We tested airway reactivity to Ach, lung pathology, levels of Th2 cytokines in the BALF, and NOS expression and NO production in lung tissues. We compared these effects to those seen in OVA-treated rats receiving systemic administration of ketamine via the intraperitoneal (i.p.) injection, and lastly tested the effects of different concentrations of nebulized ketamine on lung tissues and plasma levels in normal, non-allergic rats.

## Methods

### Sensitization and challenge protocol

Specific pathogen-free, inbred male Brown-Norway rats (strain BN/CrlBR), 7–11 weeks old and weighing between 130–170 g, were purchased from Beijing Vitalriver Laboratory Animal Co. (Beijing, China) and maintained in SPF rooms in an animal facility of the Nanjing Medical University (Jiangsu Province, China, SYXK 2002-0013). All animal care protocols and experimental procedures were performed according to institutional guidelines and conformed to the requirements of the state authority for animal research conduct.

Rats were randomly assigned to ten experimental groups (n = 6–8 rats), and the sensitization and challenge protocols were performed according to Elwood et al. [17] and Vanacker et al. [18], with some modifications, as described below.

On days (d) 0 and 7, rats were activily sensitized by subcutaneous injection of 1 mg OVA(Grade V, Sigma-aldrich, Inc. Missouri, USA) and 100 mg aluminum hydroxide [Al(OH)_3_; Jingshan Chemicals Co., Shanghai, China] in 1 ml PBS. Simultaneously, each rat received an intraperitoneal injection of 1 ml Bordetella pertussis (National Institute for the Control of Pharmaceutical and Biological Products, Beijing, China) containing 6 × 10^9 ^heat-killed bacilli as an adjuvant.

#### PBS control group (sensitized/PBS-exposed rats, n = 8)

From d 13 to d 26, the sensitized rats were exposed to aerosolized PBS for 30 minutes each day.

#### OVA control group (sensitized/PBS-exposed/OVA-challenged rats, n = 8)

Sensitized rats were exposed to aerosolized PBS for 30 min per day on odd days from d 13 to d 25, and challenged with 10 mg/ml OVA (1 g OVA in 100 ml sterile PBS in the nebulizer) for 30 min per day on even days from d 14 to d 26.

#### 12.5 mg/ml inhaled test group (sensitized/12.5 mg/ml ketamine-exposed/OVA-challenged rats, n = 8)

Sensitized rats were exposed to 12.5 mg/ml ketamine [100 mg/2 ml Ketamine Hydrochloride Injection (H32025255; Jiangsu HengRui Medicine Co., China) diluted to 12.5 mg/ml with 6 ml sterile PBS in the nebulizer] aerosol for 30 min per day on odd days from d 13 to d 25, and challenged with 10 mg/ml OVA for 30 min per day on even days from d 14 to d 26.

#### 25 mg/ml inhaled test group (sensitized/25 mg/ml ketamine-exposed/OVA-challenged rats, n = 8)

Sensitized rats were exposed to 25 mg/ml ketamine (100 mg/2 ml Ketamine Hydrochloride Injection diluted to 25 mg/ml with 2 ml sterile PBS in the nebulizer) aerosol for 30 min per day on odd days from d 13 to d 25, and challenged with 10 mg/ml OVA for 30 min per day on even days from d 14 to d 26.

#### 50 mg/ml inhaled test group (sensitized/50 mg/ml ketamine-exposed/OVA-challenged rats, n = 8)

Sensitized rats were exposed to 50 mg/ml ketamine (100 mg/2 ml Ketamine Hydrochloride Injection in the nebulizer) aerosol for 30 min per day on odd days from d 13 to d 25, and challenged with 10 mg/ml OVA for 30 min per day on even days from d 14 to d 26.

#### 50 μg/kg injected comparison group (sensitized/50 μg/kg ketamine i.p.-injected/OVA-challenged rats, n = 6)

Rats were sensitized, and then 14 days later given i.p. injections of 50 μg/kg ketamine (100 mg/2 ml Ketamine Hydrochloride Injection). The rats were challenged with 10 mg/ml OVA for 30 min at 60 min post-i.p. injection, and on even days from d 14 to d 26.

#### 100 μg/kg injected comparison group (sensitized/100 μg/kg ketamine i.p.-injected/OVA-challenged rats, n = 6)

Rats were sensitized, and then 14 days later given i.p. injections of 100 μg/kg ketamine (100 mg/2 ml Ketamine Hydrochloride Injection). The rats were challenged with 10 mg/ml OVA for 30 min at 60 min post-challenged injection, and on even days from d 14 to d 26.

The effects of nebulized ketamine at different concentrations on the normal lung structure and the plasma levels were evaluated by exposure non-sensitized rats to aerosol of ketamine at 12.5 mg/ml or 25 mg/ml or 50 mg/ml respectively for 30 min once every 2 d from d 13 to d 26.

Aerosol exposure was accomplished by placing 3–4 rats from the same group in a plexiglass chamber (30 × 30 × 30 cm^3^) connected to an ultrasonic nebulizer (S-888E, Sino-Foreign, Dolphin Nanjing Electronics Co., China), which generated an aerosol mist (particle size approximately 2–4 μm) with a drive flow rate of 5 liters/min according to specifications of the manufacturer.

### Assessment of airway responsiveness in vivo

Airway reactivity to Ach was assessed in vivo 16–18 hr after the last OVA challenge, as previously described [19]. Rats were anesthetized with pentobarbital sodium (100 mg/kg i.p.), a tracheal cannula was inserted via tracheotomy for mechanical ventilation, and a small catheter (22G) was inserted into the external jugular vein for Ach administration. The rat was then placed in a sealed whole body plethysmograph and connected to a rodent ventilator (ML-V2, Peking SYNOL High-Tech Co., Beijing, China), and ambient air was administered with a tidal volume of 8 ml/kg and a frequency of 80 strokes per minute. Transducers (ML-AMP II, Peking SYNOL High-Tech Co., Beijing, China) connected to the ventilatory circuit provided voltage signals of pressure and flow, which were amplified and transmitted to the analog/digital (A/D) card (National Instruments Texas, USA) of a microcomputer running the AniRes2003 software (Peking Biolab Tech Co., Beijing, China), which was used to calculate the inspiratory resistance (R_i_), expiratory resistance (R_e_) and dynamic compliance (Cldyn) of the respiratory system from the digitized pressure and flow signals. After stabilization of respiratory parameters (10–15 min), the rat was given Ach (dissolved in 0.9% NaCl) intravenously at an initial dose of 12.5 μg/kg, with the dose increasing two-fold each injection up to 200 μg/kg, to obtain a response curve of lung resistance (R_L_) increase over baseline. The typical injection volume was 100 μl, delivered over 3–4 s, and injections were administered at intervals of ~5–6 minutes, based on the criterion that the R_L _must return to the pre-Ach level before initiation of the next Ach administration. The response was measured as the peak increase above the baseline immediately after Ach administration.

### Bronchoalveolar lavage

After assessment of airway reactivity, the rats were bled and sacrificed under overdose anesthetic. Lungs were lavaged via the tracheal cannula with three times, once with 1 ml and twice with 1.5 ml (using sterile PBS in both cases). Lavage fluid was recovered by gentle manual aspiration with a syringe. The retrieved volume, which averaged 75–80% of the instilled PBS, was immediately centrifuged (500 *g *× 10 min at 4°C) and the supernatant was stored at -20°C until it was used for measurement of IL-4 and IL-13 levels. The pellet was kept on ice, washed twice with PBS and resuspended in 1 ml of PBS. Total numbers of leukocytes in the bronchoalveolar lavage fluid (BALF) were determined with a Coulter counter(Coulter Electronics Ltd., Harpeneden, UK). A differential cell count was performed on Cytospin (Thermo Shandon, Pittsburgh, PA) by Wright – Giemsa staining.

### Histological examination of lung sections

Following sacrifice, the right main bronchus of each rat was immediately separated and ligated, and the right lungs were removed and rinsed with diethylpyrocarbonate (DEPC)-treated water. The lung tissues were divided into several portions, frozen in liquid nitrogen, and stored at -70°C until use for RT-PCR and Western blot analysis. A cannula was inserted into the pulmonary artery via the right ventricle, and the left lung of each rat was perfused *in situ*, with fluids draining to the left atrium outflow, above the level of the atrium. Initially, the lungs were perfused with 0.9% NaCl (with a dilution 1:10000 of heparin) in an open, nonrecirculating mode at a rate of 8 ml/min for 10 min, for removal of residual blood. Next, the lungs were fixed by perfusion at 4 ml/min with 4% paraformaldehyde in 0.1 M PBS for 50 min. After perfusion, lung tissue samples were taken from peripheral areas containing alveoli and microvessels, from central lung areas containing mainstem bronchi, and from midlung areas containing a mix of peripheral tissue and small airways. These samples were stored in 4% paraformaldehyde, embedded in paraffin, sliced into 5 μm-thick sections and stained with hematoxylin-eosin. The histological sections were examined by semiautomatic morphometry using the LEICA Qwin 2.6 Image Processing and Analysis System (Leica Cambridge, Cambridge, England) coupled to a LEICA Qwin DC 300F digital camera. The lung sections were assigned a score of 0–4 (0, no inflammation; 1, mild inflammation; 2, moderate inflammation; 3, severe inflammation; 4, extreme inflammation), as previously described by Stenton et al. [20]. Inflammation was scored by a pathologist who was blind to the various treatments, and the inflammatory scores were based on the presence of congestion, hemorrhage, edema (alveolar and interstitial), and/or inflammation (airway lumen, airway wall, alveolar, interstitial, and perivascular).

### Assay of IL-4/IL-13 concentration in BALF

The supernatant obtained from the BALF was assayed for interleukin-4 (IL-4) and interleukin-13 (IL-13) concentration within one month of processing, using the enzyme-linked immunosorbent assay (ELISA)-based Quantikine M kits specific for rat IL-4 and IL-13 (R&D Systems, Minneapolis, USA), according to the manufacturer's instructions. Each kit had a sensitivity of < 5 pg/ml. The polynomial equation (y = a + bc + cx^2^) was used to create a curve for standards containing known concentrations of each cytokine, and the IL-4 and IL-13 concentrations in the BALF samples were calculated from the line equation and the sample dilution factor.

### RT-PCR analysis of Nitric Oxide Synthase (NOS) mRNA expression

Total RNA was extracted from lung tissues using the Trizol Reagent (Invitrogen, Carlsbad, CA), according to the manufacturer's protocol. Reverse transcription (RT) was performed with oligo(dT) using Superscript II RNA-reverse transcriptase (Invitrogen, Carlsbad, CA). First-strand cDNA synthesis was performed at 42°C for 1 h, the RNA-cDNA hybrids were denatured at 90°C for 10 min, and the RT products were stored at -20°C until use. PCR was performed using a Hybaid PCR Express thermal cycler (Peltier Thermal Cycler-200; MJ Research Inc., Massachussets, USA).

Primer sequences were as follows: iNOS, 5'-TTGGAGCGAGTTGTGGATTG-3' (sense) and 5'-GTGAGGGCTTGCCTGAGTGA-3' (antisense); eNOS, 5'-AGACCGATTACACGACATTGAG-3' (sense) and 5'-GACATCACCGCAGACAAACA-3' (antisense); nNOS, 5'-AGAGGAGGACGCTGGTGTA-3' (sense) and 5'-GGCGGTTGGTCACTTCATA-3' (antisense); β-actin, 5'-ATGGTGGGTATGGGTCAGAAGG-3' (sense) and 5'-CATGGCAGAAGAAAGACAATTA-3' (antisense).

The predicted sizes for the PCR products were 104 bp for nNOS, 125 bp for iNOS, 274 bp for eNOS, and 315 bp for β-actin. The PCR reactions consisted of 0.3 μL of each primer, 2 μL of dNTP, 0.2 μL of Taq DNA polymerase and 4 μL of cDNA in a final volume of 20 μL. The PCR conditions were as follows: 94°C for 5 min, followed by 32 cycles of denaturation at 94°C for 45s, annealing at 63°C (iNOS), 61°C (eNOS) or 59°C (nNOS) for 45s, and extension at 72°C for 45s, followed by a final extension at 72° for 10 min. β-Actin, which was used as an internal control, was amplified under the following conditions: 94°C for 3 min, followed by 28 cycles of 94°C for 30s, 58°C for 30s, 72°C for 30s and 72° for 10 min. The reaction products were resolved by agarose gel electrophoresis, stained with ethidium bromide (10 mg/ml), visualized under UV transillumination and photographed with a Canon TV Zoom Lens (Canon, Tokyo, Japan) in an Epi Chemi II Darkroom (UVP Laboratory Products, Upland, CA). Relative mRNA expression was estimated by comparison to the relative intensities of known DNA standards and β-actin.

### Measurement of iNOS protein levels by Western Blot

For Western Blotting, 180–220 mg of pulmonary tissue was placed in 1 ml of lysis buffer (50 mM Tris-HCl, pH 7.5, 0.3 M NaCl, 1% NP-40, 0.1% SDS, 1 mM EDTA, 10 μg/ml aprotinin, 100 μg/ml leupeptin, 1 mmol/L PMSF) and homogenized on ice using an homogenizer (IKA-ULTRA-TURRAX T25, IKA-WERKE GMBH & CO., KG, Germany) for four bursts of 10–15 s at 20 s intervals. The homogenate was centrifuged at 14,000 g for 10 min at 4°C (5810R, Eppendorf AG 22331; Eppendorf, Hamburg, Germany), the supernatant was collected, and the protein concentration was determined by the Bradford assay. Aliquots containing ~30 μg of protein were resolved in a 40 mA system(Bio-Rad, Hercules, CA, USA) consisting of an 8% polyacrylamide resolving gel and a 5% polyacrylamide stacking gel. The resolved proteins were electrophoretically transferred to PVDF membranes at 100 V for 1 hr. The membranes were washed in PBS buffer (pH 7.4) containing 0.1% (v/v) Tween-20 (PBST), blocked with 5% BSA/PBST (m/v) for 1 hour at 37°C, and then incubated overnight at 4°C with monoclonal anti-iNOS antibodies (N9657; Sigma-aldrich, Inc. Missouri, USA) diluted 1:1000 in 5% BSA/PBST. The membranes were then washed three times (5 min each) with PBST and incubated for 1 hour with horseradish peroxidase-labeled goat anti-mouse IgG diluted 1:2000 in 5% BSA/PBST. Immunoreactive bands were detected by chemiluminescence (Phototope^®^-HRP Western Blot Detection System; Cell Signaling, UK), and the results were visualized by autoradiography, photographed with a Canon TV Zoom Lens in an Epi Chemi II Darkroom, and analyzed for the relative intensity of the iNOS signal versus that of β-actin, using the Gel-Pro Analyzer software (Media Cybernetics Inc., Georgia, USA).

### Assay of Nitric Oxide (NO) production

Nitric oxide production was determined by colorimetric measurement of nitrite, which is the stable end product of nitric oxide metabolism. Briefly, pulmonary tissues were homogenized in buffer (1/10, w/v) containing 0.05 mol/L Tris-HCl, pH 7.5, 0.025 mol/L sucrose, and 0.1 mmol/L EDTA. Then this lysate was centrifuged at 1500 g for 10 min. The supernatant or a sodium nitrite standard (100 μL each) was reacted with an equal volume of Griess reagent (1% sulphanilamide, 0.1% N-(1-Napthyl) Ethylenediamine dihydrochloride, 2.5% phosphoric acid; Griess Reagent Kit, JianCheng, Jiangsu, China) in duplicate wells of a microtiter plate for 10 min at room temperature. Chromophore absorbance at 550 nm was determined using a microplate reader (Beckman Coulter, Germany). Equation is established by running known concentrations of sodium nitrite and then plotting absorbance vs. concentration. The concentration of nitrite in the samples was calculated by comparison to the standard curve of sodium nitrite.

### Determination of plasma ketamine concentration

After the last administration of nebulized ketamine by inhalation, blood samples of ~0.5 ml were collected at 0, 5, 10, 20, 40 and 60 min post-dosing from rats in 12.5 mg/ml, 25 mg/ml, and 50 mg/ml ketamine inhalation control groups respectively. The rats were then killed by overdose anesthetic, and processed for histological examination. Another blood sample was collected from a naive Brown-Norway rat as blank plasma for ketamine standard. The blood samples were centrifuged at 1500 rpm for 10 min, and the plasma was stored at -20°C until analysis.

Ketamine(white crystalline powder, pure degree 99. 8 %, 05051112, Jiangsu Hengrui Medicine Co., China) was dissolved in methanol to prepare standard stock solutions at concentrations of 5, 0.5 and 0.1 g/L and phenacetin (internal calibrator) was dissolved in methanol at 10 mg/L to make standard stock solution. These solutions were stored at -80°C. Prior to experiments, ketamine standard stock solutions were used to make standard working solutions of the appropriate dilutions with blank plasma at serials of concentrations of 250, 500, 2000, 10000 μg/L.

Ketamine levels in the plasma samples were measured by high performance liquid chromatography (HPLC) with fluorescent detection [21] using the system of Prominence Series Modular HPLC (Shimadzu Scientific Instruments, Kyoto, Japan). Separation was achieved using a Diarnosil C_18 _column (5 μm, 4.6 mm × 250 mm). The mobile phase consisted of a 65:35 (v/v) mix of acetonitrile and phosphate buffer (an equal mixture of 0.025 mol/L K_2_HPO4 and 0.003 mol/L KH_2_PO4) adjusted to pH 7.2. The flow rate of the mobile phase was 1.0 ml/min, the detection wavelength was 220 nm, and the system was used at ambient temperature (~30°C). For plasma preparation, ~0.2 ml sample plasma or 0.2 ml of ketamine standard series was mixed with 20 μL of internal calibrator, alkalinized with 20 μL of 0.2 mol/L NaOH, and vortexed for 10 min at 60 rpm, then extracted with 2 ml of a mixture containing N-hexane, lsopropylalcohol and dichloromethane (64:3:33 by vol). The mixture was centrifuged at 3500 rpm for 10 min at 15°C, and the organic layer was transferred to a conical glass tube and evaporated to dryness under a gentle nitrogen stream. Finally, the dried residue was reconstituted in 100 μL of mobile phase, and an aliquot of 20 μL was injected into the C_18 _column.

Calibration curves containing an internal correction standard were fitted by plotting the peak height ratio of the ketamine to the phenacetin vs. the known concentration of ketamine standard solutions. The concentration of ketamine in the samples was calculated on calibration curves of ketamine standard.

### Data analysis

All statistical analyses were performed using the SPSS software (version 13.0 for Windows, Northwestern University Information Technology, Evanston). Comparisons were carried out using ANOVA and Dunnett's test. The log transformation linear regression U-test was used to examine pair-wise differences in concentration under same % increase of Re, and the post-hoc Bonferroni method was used to adjust the resulting *P*-values. The presented data are expressed as the mean ± the standard error of the mean (SEM). Differences were considered statistically significant at *P *< 0.05.

## Results

### Effects of ketamine on ovalbumin-induced airway hyperresponsiveness

**Figure 1:**
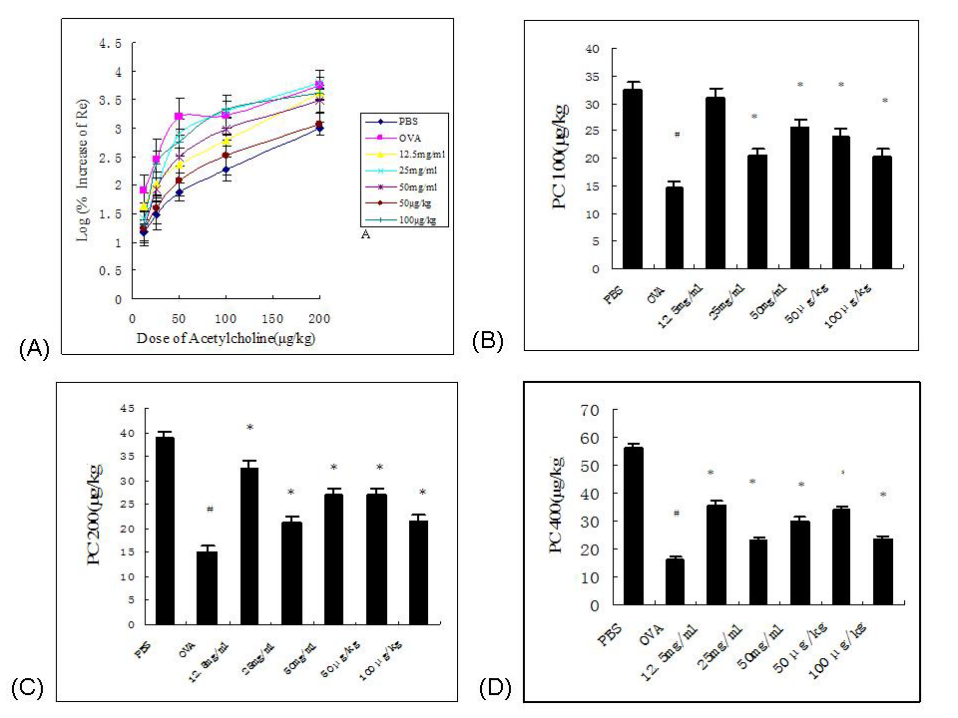
**Effects of ketamine on ovalbumin-induced airway hyperresponsiveness in vivo**. Airway reactivity to intravenous acetylcholine (Ach) was measured 16–18 hr after the last challenge, and is given as the increase in expiratory resistance (Re). (A) Responses were calculated as log percentages of the maximum Re increase above baseline following administration of different concentrations of Ach. (B, C, D) PC100, PC200 and PC400 represent the provocation concentration required to increase Re by 100, 200 and 400%, respectively. The log transformation linear regression U-test was used to examine pair-wise differences in concentration under the same % increase of Re above baseline, with the post-hoc Bonferroni method used to adjust the *P*-values. The data presented are given as the mean ± SEM (*n *= 6–8). # *P *< 0.05 vs. PBS control, * *P *< 0.05 vs. OVA control. PBS, negative control; OVA, positive control; 12.5 mg/ml, OVA-sensitized/12.5 mg/ml nebulized ketamine-exposed/OVA-challenged; 25 mg/ml, OVA-sensitized/25 mg/ml nebulized ketamine-exposed/OVA-challenged; 50 mg/ml, OVA-sensitized/50 mg/ml nebulized ketamine-exposed/OVA-challenged; 50 μg/kg, OVA-sensitized/50 μg/kg ketamine-injected i.p./OVA-challenged; 100 μg/kg, OVA-sensitized/100 μg/kg ketamine-injected i.p./OVA-challenged.

In response to increasing doses of intravenously administered Ach, all experimental rats showed dose-dependent increases in Re. There was no statistically significant difference in baseline value of Re among all experimental groups (data not shown). The airway responsiveness to Ach increased in OVA control group when compared with that of PBS-exposed animals. This is illustrated by a left-ward shift of the dose-response curve (Figure 1A). In addition, the provocation doses required to increase Re by 100, 200 and 400 % in OVA control rats were significantly lower than those of PBS control rats (14.65 ± 1.19 μg/kg vs. 32.28 ± 1.43 μg/kg, *P *< 0.001; 15.17 ± 1.19 μg/kg vs. 38.91 ± 1.39 μg/kg, *P *< 0.001 and 16.28 ± 1.18 μg/kg vs. 56.53 ± 1.38 μg/kg, *P *< 0.001, respectively; Figures 1B, C, D). Treatment with ketamine either via inhalation or injection prior to OVA challenge in OVA-sensitized rats prevented this increase in airway reactivity, reflected by right-ward shifts of the dose-response curves and significant increases in the provocation doses, when compared with OVA control rats. In groups receiving 12.5, 25 and 50 mg/ml ketamine by inhalation and 50 and 100 μg/kg ketamine by i.p. injection, the doses required to increase the Re by 100% (PC100) were 31.09 ± 1.51 μg/kg (*P *< 0.001), 20.44 ± 1.28 μg/kg (*P *= 0.019), 25.57 ± 1.38 μg/kg (*P *< 0.001), 24.02 ± 1.32 μg/kg (*P *< 0.001) and 20.39 ± 1.29 μg/kg (*P *= 0.022) respectively. Similarly, the values of PC200 and PC400 in all ketamine pretreatment groups were significantly higher than those of OVA control rats(*P *< 0.001).

### Effects of ketamine on ovalbumin-induced airway inflammation

#### Inflammatory cell infiltrate in BALF

**Figure 2:**
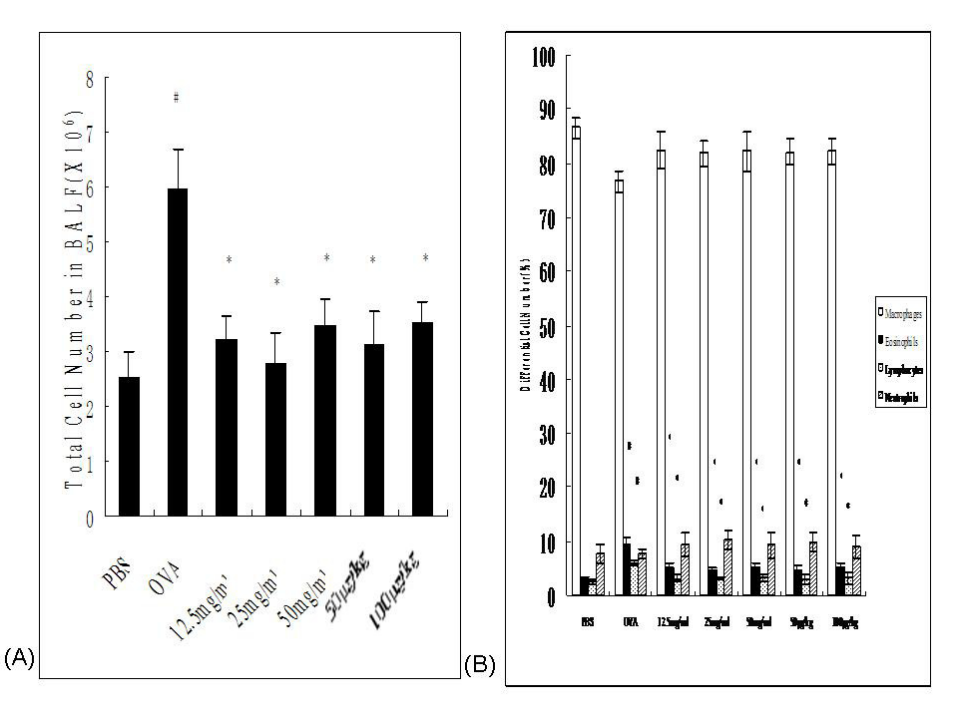
**Effects of ketamine treatment on changes in total and differential cell numbers in BALF from ovalbumin-sensitized and -challenged rats**. The total (A) and differential (B) cell numbers were counted in BALF from rats in the various treatment groups. Bars represent mean ± SEM (n = 6–8/group). # *P *< 0.05 vs. PBS control, * *P *< 0.05 vs. OVA control. The groups are as shown in Figure 1.

The majority of cells identified in the BALF of rats were macrophages (approximate 76–86 %), with smaller populations of eosinophils, neutrophils, and lymphocytes observed. Remarkably, the total cellularity of BALF recovered from OVA control rats showed significantly higher than from PBS-exposed rats (5.97 ± 0.72 × 10^6 ^vs. 2.51 ± 0.49 × 10^6^, *P *< 0.001; Figure 2A). Differential cell counting revealed a significantly higher percentage of eosinophils and lymphocytes and a decrease in the percentage of macrophages in OVA control animals compared with these parameters in PBS control animals (*P *< 0.001, *P *= 0.002 and *P *= 0.034, respectively; Figure 2B). Treatment with 12.5, 25 and 50 mg/ml nebulized ketamine or 50 and 100 μg/kg i.p.-injected ketamine inhibited OVA-induced increases in total BALF cell number by 46, 53, 41, 48 and 41% respectively, compared with OVA control animals (Figure 2A). The proportions of eosinophils in BALF from 12.5, 25 and 50 mg/ml inhaled ketamine-treated rats were 5.16 ± 1.07, 4.54 ± 0.78 and 5.31 ± 0.91%, respectively, and those in 50 and 100 μg/kg i.p.-injected ketamine-treated rats were 4.92 ± 0.66 and 5.02 ± 0.91%, compared with 9.56 ± 0.99% in OVA control animals (*P *= 0.002, *P *< 0.001, *P *= 0.004, *P *= 0.003 and *P *= 0.004 respectively). Moreover, ketamine pretreatment prior to OVA challenge rescued the percentages of lymphocytes in the BALF of OVA-sensitized rats to 3.15 ± 0.62, 3.25 ± 0.28, 3.22 ± 0.73, 3.08 ± 0.91 and 3.36 ± 1.10 %, compared with 6.08 ± 0.56 % in OVA-sensitized and -challenged rats (*P *= 0.01, *P *= 0.014, *P *= 0.013, *P *= 0.016, *P *= 0.035 respectively; Figure 2B).

#### Lung histology

**Figure 3:**
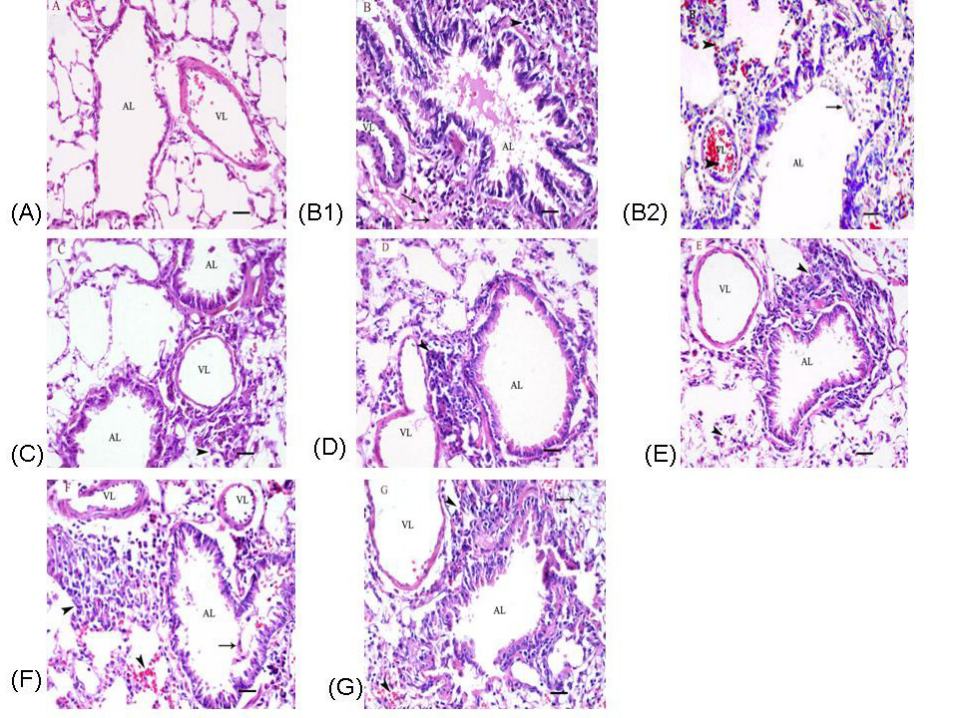
**Effects of ketamine on histopathological changes seen in pulmonary sections from ovalbumin-sensitized and -challenged rats**. Representative paraffin-embedded, Hematoxylin and Eosin-stained lung sections were prepared from the left lungs of experimental rats, showing bronchioles, lung alveoli and surrounding vessel structures. The groups are as shown in Figure 1. (A) PBS; (B1-B2) OVA; (C) 12.5 mg/ml; (D) 25 mg/ml; (E) 50 mg/ml; (F) 50 μg/kg; (G) 100 μg/kg. VL, vascular lumen; AL, airway lumen. Arrows demonstrate marked perivascular edema (B1, G), places where inflammation has destroyed a portion of the airway epithelium (B2, F), and eosinophils (B1, C, E). Arrowheads indicate peribronchial inflammation (B1, C, D, E, F, G), hemorrhage (B2, E, F, G) and congestion (B2). Sections were evaluated under light microscopy. Original magnification was ×*40*, and the scal bars represent 20 μM.

In PBS-treated rats, the small bronchi, bronchioles and lung alveoli were structurally normal, the mucosal epithelia were intact, and no inflammation was present (Figure 3A). Conversely, the airways of OVA control rats showed marked inflammatory changes, including desquamation of the bronchial epithelia, the presence of secretions and damaged cells inside the lumens of bronchi and alveoli, and patchy inflammatory infiltration in the bronchial submucosa, perivascular areas and the surrounding alveolar septa. These infiltrates consisted primarily of mononuclear cells and some eosinophils. In addition, we observed that OVA exposure induced goblet cell hyperplasia, hemorrhage, congestion, and alveolar and interstitial edema (Figures 3B1, B2). Notably, ketamine-treated rats showed less infiltration of inflammatory cells into peribronchial and perialveolar areas, decreased interstitial edema, and fewer epithelial lesions in the bronchi and bronchioles, although the goblet cell hyperplasia and congestion triggered by OVA exposure did not appear to be affected by ketamine treatment (Figures 3C–G).

**Figure 4:**
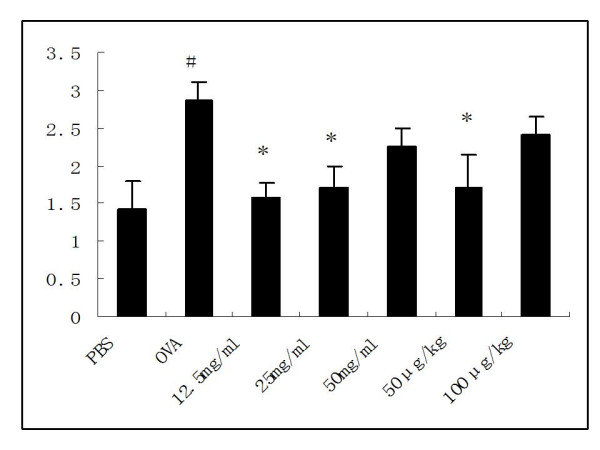
**Effects of ketamine on inflammatory scores in lung tissues from ovalbumin-sensitized and -challenged rats**. Lungs were sampled after measurement of airway reactivity and scored under the light microscope at a magnification of ×40. Bars represent mean ± SEM (n = 6–8/group). # *P *< 0.05 vs. PBS control, * P < 0.05 vs. OVA control. The groups are as shown in Figure 1.

The mean inflammatory score significantly increased from 1.43 ± 0.37 in PBS-exposed rats to 2.88 ± 0.23 in sensitized rats challenged with OVA (P = 0.003). When compared with OVA control animals, inflammatory scores were significantly decreased at the level of 12.5 or 25 mg/ml nebulized ketamine or 50 μg/kg i.p.-injected ketamine 1.57 ± 0.2 (*P *= 0.009), 1.71 ± 0.29(*P *= 0.023), 1.7 ± 0.44(*P *= 0.043) respectively, while the dose of 50 mg/ml nebulized ketamine or 100 μg/kg i.p.-injected ketamine did not show statistical signifiance(Figure 4). There was no statistically significant difference in pulmonary inflammatory scores and the changes of lung structure among PBS-treated controls and non-sensitized rats receiving inhaled ketamine at concentrations of 12.5, 25 or 50 mg/ml (data not shown).

#### Levels of IL-4 and IL-13 in BALF

**Figure 5:**
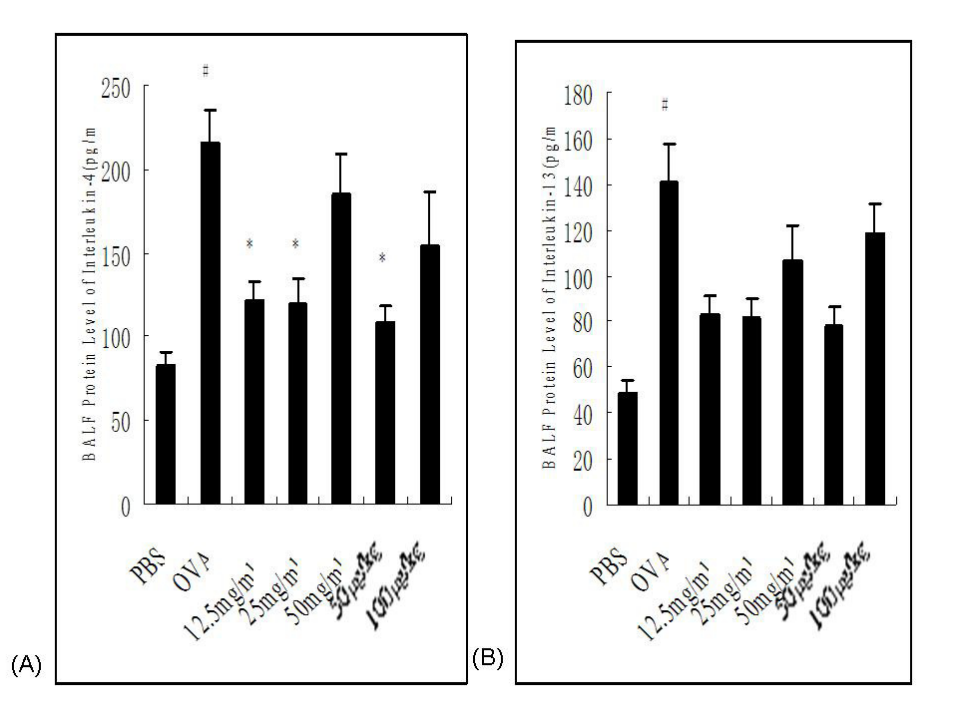
**Effects of ketamine on Interleukin-4 and Interleukin-13 protein levels in BALF from ovalbumin-sensitized and -challenged rats**. IL-4 (A) and IL-13 (B) protein levels in BALF were measured by ELISA. Bars represent mean ± SEM (n = 6–8/group). # *P *< 0.05 vs. PBS control, * P < 0.05 vs. OVA control. The groups are as shown in Figure 1.

BALF from PBS control rats contained 82.81 ± 7.87 pg/ml of IL-4, whereas this level was 2- to 3-fold higher in OVA control animals with a mean concentration of 215.33 ± 20.75 pg/ml (*P *= 0.004). OVA-sensitized and -challenged rats treated with 12.5 or 25 mg/ml nebulized ketamine or 50 μg/kg i.p.-injected ketamine showed significant decreases in IL-4 protein levels compared to OVA control animals (121.30 ± 11.18 pg/ml, *P *= 0.036; 119.33 ± 14.60 pg/ml, *P *= 0.041; 108.60 ± 9.34, *P *= 0.016, respectively). There was no statistical difference in IL-4 levels among rats receiving 50 mg/ml inhaled ketamine or 100 μg/kg i.p.-injected ketamine and OVA control rats (Figure 5A). In terms of IL-13, OVA-sensitized/PBS-exposed/OVA-challenged animals had significantly higher IL-13 levels (140.73 ± 16.47 pg/ml) compared to PBS-challenged rats (48.40 ± 5.57 pg/ml, *P *= 0.009). Treatment with inhaled or injected ketamine appeared to decrease the IL-13 levels in BALF, but these differences did not reach the level of statistical significance(Figure 5B).

#### NOS mRNA expression in pulmonary tissues

**Figure 6:**
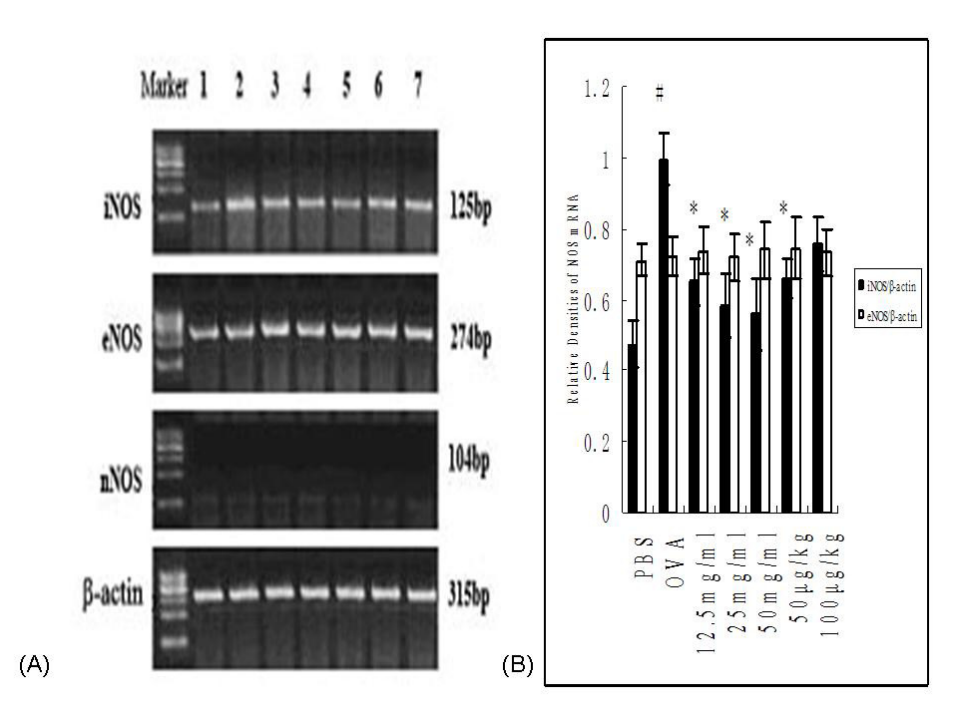
**Effects of ketamine on NOS mRNA expression in pulmonary tissues from ovalbumin-sensitized and -challenged rats**. (A) Expression of NOS mRNA in pulmonary tissues was detected by RT-PCR. Representative RT-PCR products for nNOS, iNOS, eNOS and β-actin (internal control) are shown. The groups are as shown in Figure 1. *Lane 1*, PBS; *lane 2*, OVA; *lane 3*, 12.5 mg/ml; *lane 4*, 25 mg/ml; *lane 5*, 50 mg/ml; *lane 6*, 50 μg/kg; *lane 7*, 100 μg/kg. (B) Semiquantitative densitometry was used to analyze iNOS and eNOS mRNA expression levels, normalized to that of β-actin. Bars represent mean ± SEM expressed as the relative density of NOS versus that of β-actin (n = 6–8/group). ^# ^P < 0.05 vs. PBS control; * P < 0.05 vs. OVA control.

RT-PCR was used to examine the expression of nNOS, iNOS and eNOS in pulmonary tissues from rats in all treatment groups. Expression of iNOS and eNOS was detected in the studied samples, but nNOS was not (Figure 6A). The internal control housekeeping gene, β-actin, was used to calculate relative mRNA expression levels. We found that iNOS mRNA expression was significantly higher in OVA controls versus PBS controls (1.00 ± 0.07 vs. 0.48 ± 0.07, *P *< 0.001). Ketamine treatment significantly decreased iNOS mRNA expression in OVA-sensitized and -challenged rats receiving 12.5 (0.65 ± 0.07, *P *= 0.01), 25 (0.58 ± 0.09, *P *= 0.002) or 50 (0.56 ± 0.100, *P *= 0.001) mg/ml inhaled ketamine, or 50 μg/kg i.p.-injected ketamine (0.66 ± 0.06, *P *= 0.028) respectively. In contrast, there was no significant difference in eNOS mRNA expression levels among the various experimental groups (Figure 6B).

#### iNOS protein expression in pulmonary tissues

**Figure 7:**
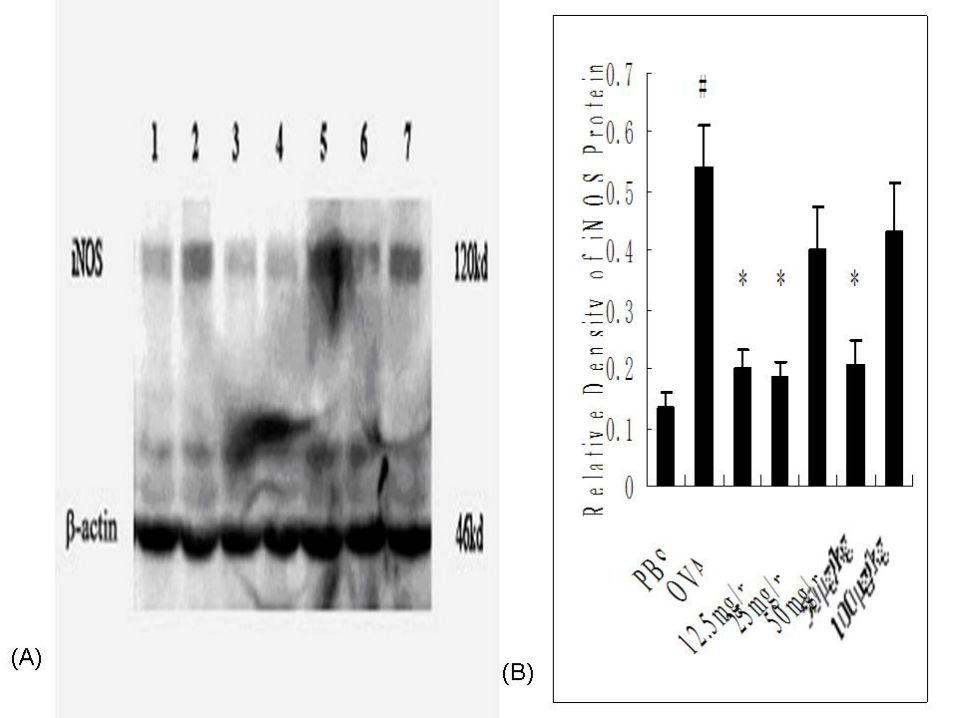
**Effects of ketamine on iNOS protein expression in pulmonary tissues from ovalbumin-sensitized and -challenged rats**. Protein expression of iNOS in lung homogenates obtained from experimental rats was examined by Western blotting, with a representative example from each group shown. β-actin was detected as an internal control. The groups are as shown in Figure 1. (A) *Lane 1*, PBS; *lane 2*, OVA; *lane 3*, 12.5 mg/ml; *lane 4*, 25 mg/ml; *lane 5*, 50 mg/ml; *lane 6*, 50 μg/kg i.p,; *lane 7*, 100 μg/kg. (B) Densitometric analysis of iNOS protein levels in lungs obtained from rats in each group. The bars represent the mean ± SEM, with laser densitometry used to standardize iNOS protein levels with respect to that of β-actin (ratio of iNOS/β-actin, n = 6–8/group). ^# ^*P *< 0.05 vs. PBS control; * P < 0.05 vs. OVA control.

Western blot analysis was used to semiquantitatively examine protein expression of iNOS (120 kDa) and β-actin (46 kDa) (Figure 7A). The relative iNOS protein levels (ratios of iNOS/β-actin) determined by densitometry showed 4-fold higher levels in OVA control rats versus PBS controls (0.54 ± 0.08 vs. 0.13 ± 0.03, *P=*0.012), whereas the OVA-induced increases were significantly lower in OVA-sensitized and -challenged rats receiving 12.5 mg/ml inhaled (0.20 ± 0.03, *P *= 0.036), 25 mg/ml inhaled (0.18 ± 0.03, *P *= 0.027), or 50 μg/kg i.p.-injected (0.21 ± 0.04, *P *= 0.045) ketamine (Figure 7B). However, 50 mg/ml inhaled or 100 μg/kg i.p.-injected ketamine did not show statistical significance.

#### NO production in pulmonary tissues

**Figure 8:**
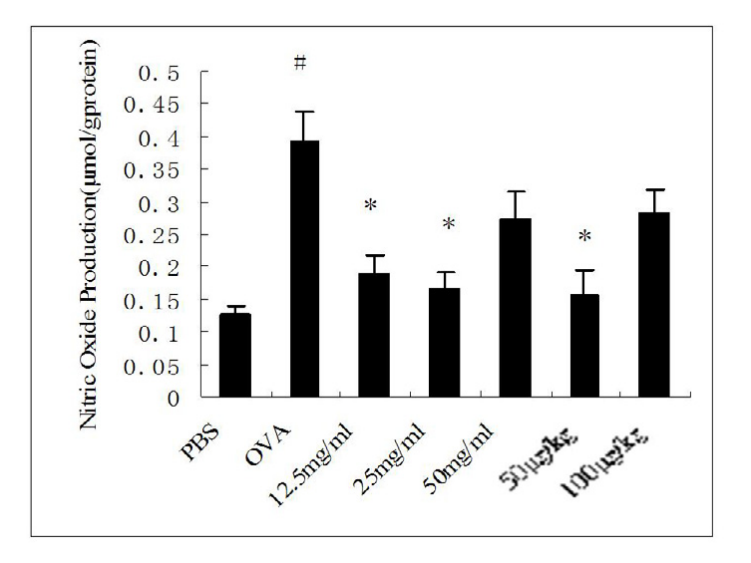
**Effects of ketamine on nitric oxide production in pulmonary tissues from ovalbumin-sensitized and -challenged rats**. Nitric oxide production was determined by measurement of nitrite, and the results are expressed as μmol/g protein. Bars represent mean ± SEM (n = 6–8/group). ^# ^P < 0.05 vs. PBS control; * P < 0.05 vs. OVA control. The groups are as shown in Figure 1.

NO production in pulmonary tissues was significantly higher in OVA control rats compared to PBS-challenged controls (0.39 ± 0.04 μmol/g protein vs. 0.13 ± 0.01 μmol/g protein, *P *= 0.005), but this OVA-triggered NO production was significantly decreased by treatment with 12.5 or 25 mg/ml inhaled ketamine (0.19 ± 0.03 μmol/g protein, *P *= 0.032; 0.17 ± 0.03 μmol/g protein, *P *= 0.014, respectively) or 50 μg/kg i.p.-injected ketamine (0.16 ± 0.04 μmol/g protein, *P *= 0.022) when compared with OVA control rats. In contrast, no significant difference in NO production was observed in OVA-sensitized and -challenged rats treated with 50 mg/ml inhaled or 100 μg/kg i.p.-injected ketamine versus OVA controls(Figure 8).

### Plasma concentration-time curves of ketamine

**Figure 9:**
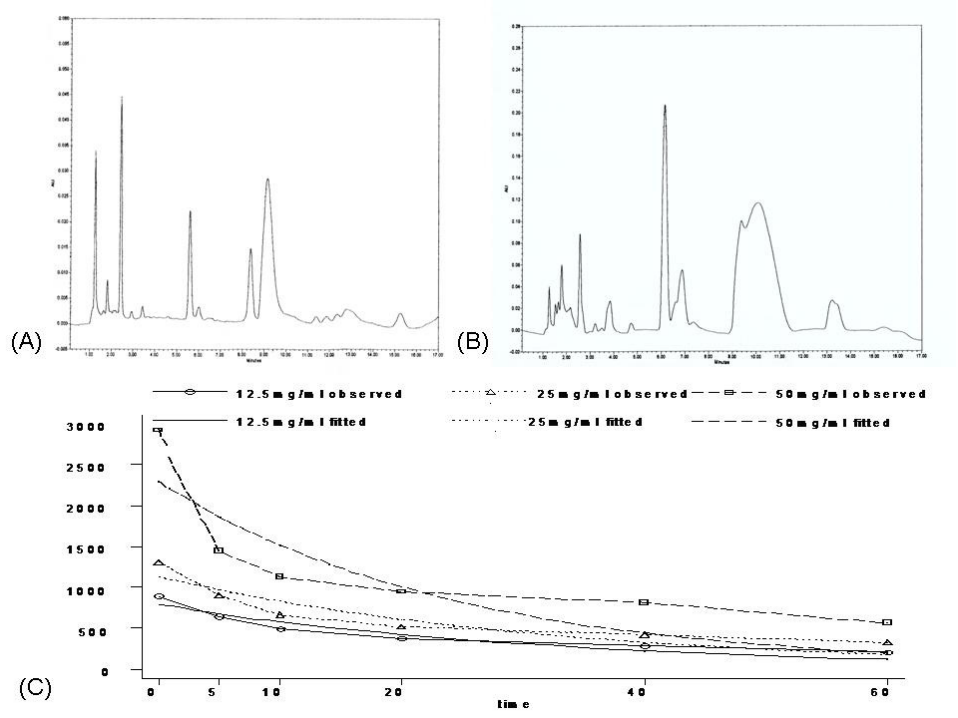
**HPLC determination of ketamine concentration in plasma from rats receiving nebulized ketamine**. The retention times of ketamine and phenacetin (internal calibrator) in the utilized system were ~5.87 and 2.58 min, respectively. (A) Chromatograms of blank plasma supplemented with 500 μg/L of a ketamine standard. (B) Representative chromatogram of plasma sample collected from a normal rat at 0 min after inhalation of 25 mg/ml ketamine. (C) Plasma concentration-time profiles of ketamine over a period of 60 min after the rats received nebulized ketamine at concentration of 12.5, 25 or 50 mg/ml respectively. The values represent the mean ± SEM (n = 6/group).

Chromatogram of a blank plasma supplemented with ketamine and phenacetin at a ketamine concentration of 500 μg/L is shown Figure 9A, and a plasma sample obtained 0 min after inhalation of 25 mg/ml ketamine is shown Figure 9B. Our results revealed that there was no detectable interference due to interactions between ketamine or phenacetin and endogenous materials in blood plasma. The separation of the ketamine and phenacetin was achieved in about 10 min. The retention times of ketamine and phenacetin in our system were determined to be about 5.87 and 2.58 min. The minimum detectable amount of ketamine at a signal-to-noise ratio of 4 was found to be 5 μg/L.

Calibration curves were found to be linear in the range of 250–10,000 μg/L ketamine (R^2 ^= 0.9984, data not shown). Plasma ketamine concentration vs. time profiles showed that the plasma concentration decreased sharply at the initial time points and then decreased more slowly after 20 min post-dosing. In rats receiving 12.5, 25 and 50 mg/ml nebulized ketamine, the plasma peak levels of ketamine were actually tested 890.33 ± 65.30, 1313.50 ± 151.65 and 2806 ± 596.14 μg/L respectively, these were occurred at 0 min. Plasma drug clearance was found to be an exponential function, and the plasma ketamine concentrations over time could be fitted to the following equations: C_fitted _= 792.68*0.97^time^(F = 106.34, P = 0.0003, R^2 ^= 0.98); C_fitted _= 1133.82*0.97^time^(F = 65.32, P = 0.0009, R^2 ^= 0.97); C_fitted _= 2288.63*0.96^time^(F = 28.08, P = 0.0044, R^2 ^= 0.93). (Figure 9C).

## Discussion

In this study, we investigated possible therapeutic effects of inhaled ketamine solution on allergic asthma, using an OVA-induced asthma model in Brown-Norway rats. We found that ketamine treatment yielded anti-inflammatory effects, as evidenced by lung histological examination, total and differential cell counts in BALF, Th2 cytokine levels in BALF, and iNOS expression and NO content in pulmonary tissues. Furthermore, the results of Ach-elicited airway response tests indicated that ketamine treatment by both inhaled and injected routes could attenuate OVA-induced AHR. Finally, inhalation of nebulized ketamine did not induce toxicological changes in lung tissues and was associated with a much lower plasma concentration, suggesting that nebulized ketamine may prove to be a safe and effective aerosol therapy for allergic asthma.

Intraperitoneal injection of ketamine was set as parallel comparison in this experiments, the aim was to observe whether the effect of nebulized ketamine on OVA-induced inflammatatory changes and AHR was as effective as that of ketamine administration by systemic route. The doses of ketamine tested by intraperitoneal injection were 50 and 100 μg/kg, with the latter corresponding to the anesthetic dose for rats. In a previous study by Rock et al. [22], four of six guinea pigs receiving 10 mg/ml aerosolized ketamine had a lower peak airway resistance compared with PBS controls, though this difference did not reach statistical significance. The authors speculated that this lack of significance might have been due to the small sample size. However, aerosol concentration may also be an important factor in evaluating the efficacy of an inhalotherapy, as the therapeutic response to an inhaled drug is a function of the drug dose that is actually deposited at the action site within the lung [3]. Therefore, based on the previous findings with 10 mg/ml inhaled ketamine, we herein examined the effect of 12.5, 25 and 50 mg/ml inhaled ketamine. Our results showed that inhalation of ketamine at 12.5 or 25 mg/ml concentration is as effective as systemic administration of ketamine at dose of 50 μg/kg for the treatment of experimental asthma *in vivo*.

The Brown-Norway rat model of allergen-induced asthma, which has been used extensively to evaluate the pathophysiological mechanisms of this disease, reflects many features of human allergic asthma, including both early and late phase reactions, increased antigen-specific IgE levels following active immunization [23], increased bronchial responsiveness to multiple stimuli following allergen challenge [24], and airway inflammation [17]. OVA sensitization alone has no effect on airway responsiveness and does not elicit measurable airway inflammation in this model [17,25]; in contrast a single OVA challenge induces transient AHR, and multiple OVA aerosol challenges trigger persistent AHR [17]. In the present study, sensitized Brown-Norway rats were subjected to repeated intermittent (thrice weekly) exposure to aerosolized OVA for two weeks. This triggered the result of increased AHR to Ach and an obvious airway inflammation observed 24 h after the last OVA challenge, which was in accordance with the report of Palmans et al. [26], confirming the co-existence of high airway reactivity and allergic inflammation in sensitized Brown-Norway rats after 2 weeks of OVA exposure.

Measurement of airway responsiveness to bronchoconstrictor stimuli continues to be advocated as a useful diagnostic test for evaluating possible asthmatics [27]. Airway responsiveness may be defined as the normal tendency for airways to constrict under the influence of various stimuli. When direct-acting bronchoconstrictors(Ach, methacholine, and carbachol) are used, hyperresponsiveness can be defined as increases in both the ease and magnitude of bronchoconstriction [27]. In our study, the leftward shift of dose-response curve corresponding to Ach-induced airway reactivity in OVA control group showed a higher magnitude of the maximal dose-response plateau under same dose of Ach, indicating an increased magnitude of bronchoconstriction. In addition, the lower values of PC100, PC200, PC400 indicating an increase in the ease of bronchoconstriction. Our experimental results demonstrated that OVA induced AHR in experimental rats, whereas this was attenuated by administration of inhaled or injected ketamine. Ketamine is a potent bronchodilator that inhibits airway smooth muscle contractions by actions occurring within both the smooth muscle cell and the vagal intramural ganglia [28]. The direct bronchorelaxing effects are likely to be achieved with the usual clinical doses in airway rings [29].

The presence of airways hyperresponsiveness and eosinophilia as a late reaction 24 h after antigen challenge of sensitized animals is well established in the literature [30,31]. Eosinophils are a major source of inflammatory mediators that can cause tissue damage and airway hyperresponsiveness in allergic asthma [32]. One hallmark of allergic asthma, namely inflammatory cell infiltration into the bronchoalveolar space, was used to evaluate the effect of different ketamine treatment regimens. Both inhalation of ketamine at concentrations of 12.5 mg/ml, 25 mg/ml, 50 mg/ml and systemic administration of ketamine at doses of 50 μg/kg and 100 μg/kg were sufficient to suppress allergen-induced inflammatory cell infiltration into the parenchyma and alveoli from the observation of lower total and eosinophil counts in BALF.

Accumulating data seem to indicate that unbalanced and aberrant Th2 inflammation is the main cause of allergic asthma. The activation of T lymphocytes and the production of cytokine mediators, leading to subsequent recruitment of effector eosinophils, may be a common pathway in the pathogenesis of asthma [33]. There is little controversy about the requirements for IL-4 in the induction of airway inflammation and AHR [34], while IL-13 direct causes AHR and mucus overproduction in asthma [35,36]. Both promote interaction of vascular cell adhesion molecule 1(VCAM-1) with the very late activation antigen 4 (VLA-4) of eosinophil activation and recruitment [37]. Previous studies *in vivo *and *in vitro *have confirmed that ketamine could reduce inflammatory cytokine production/release and inhibit certain cytokine effects [38-40]. The present study showed that ketamine treatment decreased the percentage of lymphocyte in BALF. In particular, administration of nebulized ketamine at 12.5 mg/ml and 50 mg/ml and injection of ketamine at dose of 50 μg/kg significantly down-regulate Th2 cytokine IL-4 secretion in BALF, which suggest that ketamine treatment could alleviate IL-4-mediated responses, airway inflammation and AHR.

Recent findings suggest that NO may be involved in the pathophysiology of asthma, excessive production of NO in asthma may be cytotoxic, and may also contribute to the pathologic changes (e.g. airway edema, mucus production, and inflammatory cellular infiltration) seen in patients with asthma, particularly during asthma exacerbations [41,42]. The non-invasive measurement of NO in exhaled air seems accurately to reflect inflammation in the airways and may be of value in monitoring airway diseases such as asthma. Although high NO output is poorly correlated with the degree of bronchoconstriction, this parameter correlates well with other inflammatory markers, including airway eosinophilia [43]. NO is synthesized from the conversion of L-arginine to L-citrulline by nitric oxide synthase (NOS), and at least three NOS isoforms, differing in activity and tissue distribution, have been identified. The two constitutively expressed isoforms, endothelial NOS (eNOS) and neural NOS (nNOS), both produce small (picomole) amounts of NO and mediate physiological functions in healthy lungs. In contrast, the third NOS isoform, inducible NOS (iNOS), is not expressed in normal tissues and may be induced by diverse cytokines or endotoxins, triggering extended production of larger (nanomole) amounts of NO [44]. In the present study, gene expression of eNOS and iNOS were both detected, although nNOS was not detected in the lung tissues from experimental rats. Significant differences of iNOS gene expression observed between OVA control rats and PBS-treated controls. This result was confirmed by protein expression analysis of iNOS and NO content analysis. Furthermore, ketamine pretreatment significantly reduced the OVA-triggered up-regulation of iNOS and NO levels, especially in nebulized ketamine at 12.5 or 25 mg/ml or injected ketamine at dose of 50 μg/kg. This is consistent with the observation that ketamine prevented the elevation of NO [45] and inhibited the activity and protein expression of iNOS [12,46] in response to LPS *in vitro *studies.

Our results indicated both 12.5 and 25 mg/ml inhaled ketamine and 50 μg/kg i.p.-injected ketamine exerted anti-inflammatory and anti-hyperresponsiveness roles in an established experimental model of allergic asthma. The peak plasma concentrations of ketamine in rats receiving 12.5, 25 and 50 mg/ml nebulized ketamine, as determined by HPLC, were far lower than 100 μM, which meaning that these levels were within the range of clinical relevance and without being cytotoxic to macrophages in vitro[47].

In summary, inhaled ketamine appeared to effectively block the inflammatory cascade response in an *in vivo *model of allergic asthma. Nebulized ketamine at different concentrations was found to suppress allergen-induced AHR and elevation of inflammatory markers, but this effect was not strictly dose-dependent within the scope of 12.5, 25, 50 mg/ml concentrations. Nebulized ketamine at concentrations of 12.5 or 25 mg/ml significantly lessened OVA-induced airway inflammation and the induction of iNOS, IL-4 and NO in an experimental model of asthma. This effect was ultimately accompanied by reduced airway hyperresponsiveness, suggesting that nebulized ketamine at 12.5 or 25 mg/ml might be beneficial for the treatment of asthma.

## Conclusion

Ketamine administration by local route appears to inhibit the inflammatory cascade response in an experimental asthma model *in vivo*. Inhalation of 12.5 or 25 mg/ml ketamine markedly suppressed OVA-provoked airway hyperreactivity (AHR), airway inflammation and airway inflammatory cell infiltration into BALF, and significantly decreased OVA-induced up-regulation of iNOS, IL-4 and NO. These findings collectively indicate that nebulized ketamine attenuates many of the central components of inflammatory changes and AHR in an OVA-provoked experimental asthma and may provide a new therapeutic approach for the treatment of allergic asthma.

## Competing interests

The author(s) declare that they have no competing interests.

## Authors' contributions

MMZ designed and carried out all experiments except for the PCR and HPLC. She drafted the final manuscript. QHZ assisted in performing the experiments and conceived the study. MHZ assisted in performing the experiments. HBR assisted in performing the experiments and carried out the PCR experiments. YMX assisted in performing the experiments and performed the HPLC. YNQ critically revised the manuscript. CZF supervised the study design and coordination of the experiments, and participated in drafting the manuscript.

All authors read and approved the final manuscript.

## References

[B1] Iwata A, Nishio K, Winn RK, Chi EY, Henderson WR, Harlan JM (2003). A Broad-Spectrum Caspase Inhibitor Attenuates Allergic Airway Inflammation in Murine Asthma Model. J Immunol.

[B2] Rodrigo GJ (2003). Inhaled therapy for acute adult asthma. Curr Opin Allergy Clin Immunol.

[B3] Dhand R (2000). Aerosol therapy for asthma. Curr Opin Pulm Med.

[B4] Whittaker LA, Lauren C (2002). Recent Concepts in the Pathogenesis and Treatment of Asthma [Obstructive Airways Disease]. Clin Pulm Med.

[B5] Reich DL, Silvay G (1989). Ketamine: an update on the first twenty-five years of clinical experience. Can J Anaesth.

[B6] Youssef-Ahmed MZ, Silver P, Nimkoff L, Sagy M (1996). Continuous infusion of ketamine in mechanically ventilated children with refractory bronchospasm. Intensive Care Med.

[B7] Sarma VJ (1992). Use of ketamine in acute severe asthma. Acta Anaesthesiol Scand.

[B8] Heshmati F, Zeinali MB, Noroozinia H, Abbacivash R, Mahoori A (2003). Use of ketamine in severe status asthmaticus in intensive care unit. Iran J Allergy Asthma Immunol.

[B9] Leal Filho MB, Morandin RC, de Almeida AR, Cambiucci EC, Borges G, Gontijo JA, Metze K (2005). Importance of anesthesia for the genesis of neurogenic pulmonary edema in spinal cord injury. Neurosci Lett.

[B10] Neder MT, Lazaro DSA (2004). Ketamine reduces mortality of severely burnt rats, when compared to midazolam plus fentanyl. Burns.

[B11] Sun J, Li F, Chen J, Xu J (2004). Effect of ketamine on NF-kappa B activity and TNF-alpha production in endotoxin-treated rats. Ann Clin Lab Sci.

[B12] Helmer KS, Cui Y, Dewan A, Mercer DW (2003). Ketamine/xylazine attenuates LPS-induced iNOS expression in various rat tissues. J Surg Res.

[B13] Lau TT, Zed PJ (2001). Does ketamine have a role in managing severe exacerbation of asthma in adults?. Pharmacotherapy.

[B14] Lauretti GR, Lima Izabel CPR, Reis MP, Prado WA, Pereira NL (1999). Oral Ketamine and Transdermal Nitroglycerin as Analgesic Adjuvants to Oral Morphine Therapy for Cancer Pain Management. Anesthesiology.

[B15] Garcia-Velasco P, Roman J, de Beltran HB, Metje T, Villalonga A, Vilaplana J (1998). Nasal ketamine compared with nasal midazolam in premedication in pediatrics. Rev Esp Anestesiol Reanim.

[B16] Malinovsky JM, Servin F, Cozian A, Lepage JY, Pinaud M (1996). Ketamine and norketamine plasma concentrations after iv., nasal and rectal administration in children. British Journal of Anaesthesia.

[B17] Elwood W, Lotvall JO, Barnes PJ, Chung KF (1991). Characterization of allergen-induced bronchial hyperresponsiveness and airway inflammation in actively sensitized brown-Norway rats. J Allergy Clin Immunol.

[B18] Vanacker NJ, Palmans E, Kips JC, Pauwels RA (2001). Fluticasone Inhibits But Does Not Reverse Allergen- Induced Structural Airway Changes. Am J Respir Crit Care Med.

[B19] Xu KF, Vlahos R, Messina A, Bamford TL, Bertram JF, Stewart AG (2002). Antigen-induced airway inflammation in the Brown Norway rat results in airway smooth muscle Hyperplasia. Journal of Applied Physiology.

[B20] Stenton GR, Ulanova M, Déry RE, Merani S, Kim MK, Gilchrist M, Puttagunta L, Musat-Marcu S, James D, Schreiber AD, Befus AD (2002). Inhibition of Allergic Inflammation in the Airways UsingAerosolized Antisense to Syk Kinase. J Immunol.

[B21] Bolze S, Boulieu R (1998). HPLC determination of ketamine, norketamine, and dehydronorketamine in plasma with a high-purity reversed-phase sorbent. Clinical Chemistry.

[B22] Rock MJ, Rocha SR, Lerner M, Brackett D, Wilson MF (1989). Effect on airway resistance of ketamine by aerosol in guinea pigs. Anesth Analg.

[B23] Waserman S, Olivenstein R, Renzi PM, Xu LJ, Martin JG (1992). The relationship between late asthmatic responses and antigen-specific immunoglobulin. J Allergy Clin Immunol.

[B24] Bellofiore S, Martin JG (1988). Antigen challenge of sensitised rats increases airway responsiveness to methacholine. J Appl Physiol.

[B25] Kips JC, Cuvelier CA, Pauwels RA (1992). Effect of acute and chronic antigen inhalation on airway morphology and responsiveness in actively sensitized rats. Am Rev Respir Dis.

[B26] Palmans E, Kips JC, Puuwels RA (2000). Prolonged allergen exposure induces structural airway changes in sensitized rats. Am J Respir Crit Care Med.

[B27] Cockcroft DW, Barners PJ, Grunstein MM, Leff AR, Woolcock AJ (1997). Airway Responsiveness. Asthma.

[B28] Hodgson PE, Rehder K, Hatch DJ (1995). Comparison of the pharmacodynamics of ketamine in the isolated neonatal and adult porcine airway. Br J Anaesth.

[B29] Cheng EY, Mazzeo AJ, Bosnjak ZJ, Coon RL, Kampine JP (1996). Direct relaxant effects of intravenous anesthetics on airway smooth muscle. Anesth Analg.

[B30] Cortijo J, Blesa S, Martinez-Losa M, Mata M, Seda E, Santangelo F, Morcillo EJ (2001). Effects of taurine on pulmonary responses to antigen in sensitized Brown-Norway rats. Eur J Pharmacol.

[B31] Inman MD, Ellis R, Wattie J, Denburg JA, O'Byrne PM (1999). Allergen-induced increase in airway responsiveness, airway eosinophilia, and bone-marrow eosinophil progenitors in mice. Am J Respir Cell Mol Biol.

[B32] Kay AB (2005). The role of eosinophils in the pathogenesis of asthma. Trends Mol Med.

[B33] Romagnani S (2000). The role of lymphocytes in allergic disease. J Allergy Clin Immunol.

[B34] Hamelmann E, Gelfand EW (2001). IL-5-induced airway eosinophilia-the key to asthma?. Immunological Reviews.

[B35] Kuperman DA, Huang XZ, Koth LL, Chang GH, Dolganov GM, Zho Z, Elias JA, Sheppard D, Erle DJ (2002). Direct effects of interleukin-13 on epithelial cells cause airway hyperreactivity and mucus overproduction in asthma. Nature Medicine.

[B36] Venkayya R, Lam M, Willkom M, Grunig G, Corry DB, Erle DJ (2002). Th2 Lymphocyte products IL-4 and IL-13 rapidly induce Airway Hyperresponsiveness through direct effects on resident airway cells. Am J Respir Cell Mol Biol.

[B37] Nakajima H, Sano H, Nishimura T, Yoshida S, Iwamoto I (1994). Role of vascular cell adhesion molecule 1/very late activation antigen 4 and intercellular adhesion molecule 1/lymphocyte function-associated antigen 1 interactions in antigen-induced eosinophil and T cell recruitment into the tissue. J Exp Med.

[B38] Kawasaki T, Ogata M, Kawasaki C, Ogata J, Inoue Y, Shigematsu A (1999). Ketamine suppresses proinflammatory cytokine production in human whole blood in vitro. Anesth Analg.

[B39] Takenaka I, Ogata M, Koga K, Matsumoto T, Shigematsu A (1994). Ketamine suppresses endotoxin- induced tumor necrosis factor alpha production in mice. Anesthesiology.

[B40] Hill GE, Anderson JL, Lyden ER (1998). Ketamine inhibits the proinflammatory cytokine-induced reduction of cardiac intracellular cAMP accumulation. Anesth Analg.

[B41] Ricciardolo FLM (2003). Multiple roles of nitric oxide in the airways. Thorax.

[B42] Hart CM (1999). Nitric oxide in adult lung disease. Chest.

[B43] Kharitonov SA, Yates D, Robbins RA, Logan-Sinclair R, Shinebourne EA, Barnes PJ (1994). Increased nitric oxide in exhaled air of asthmatic patients. Lancet.

[B44] Moncada S, Higgs A (1993). The L-arginine-nitric oxide pathway. N Engl J Med.

[B45] Shimaoka M, Iida T, Ohara A, Taenaka N, Mashimo T, Honda T, Yoshiya I (1996). Ketamine inhibits nitric oxide production in mouse-activated macrophage-like cells. Br J Anaesth.

[B46] Li CY, Chou TC, Wong CS, Ho ST, Wu CC, Yen MH, Ding YA (1997). Ketamine inhibits nitric oxide synthase in lipopolysaccharide-treated rat alveolar macrophages. Can J Anaesth.

[B47] Chang Y, Chen TL, Sheu JR, Chen RM (2005). Suppressive effects of ketamine on macrophage functions. Toxicology and Applied Pharmacology.

